# Continuing Professional Development—Answers

**DOI:** 10.1002/jmrs.70034

**Published:** 2025-11-21

Maximise your continuing professional development (CPD) by reading the selected article and answering the five questions. Please remember to self‐claim your CPD and retain your supporting evidence.

## Answers to Questions Published in Previous Issues

### Volume 71, Issue 4, December 2024

K. Lewis, S. Mdletshe, A. Doubleday, T. Pieterse. Preliminary image evaluation performance of radiographers in one New Zealand District: A 6‐month prospective study. *J Med Radiat Sci*. 2024 Dec; 71(4): 582–590. https://doi.org/10.1002/jmrs.810

CPD Questions https://doi.org/10.1002/jmrs.827

**Table:** 

Questions	Answers
1	D
2	A
3	B
4	A
5	D

S. Prasad, LJ. Bell, B. Zwan, et al. Comparing immobilisation devices in gynaecological external beam radiotherapy: Improving inter‐fraction reproducibility of pelvic tilt. *J Med Radiat Sci*. 2024 Dec; 71(4): 529–539. https://doi.org/10.1002/jmrs.804

CPD Questions https://doi.org/10.1002/jmrs.826

**Table:** 

Questions	Answers
1	C
2	A
3	D
4	A
5	B

### Volume 72, Issue 1, March 2025

M. Shields, D. James, L. McCormack. Occupational burnout in nuclear medicine technologists working in Australia and New Zealand—Results of a multi‐national survey. *J Med Radiat Sci*. 2025 Mar; 72(1): 25–33. https://doi.org/10.1002/jmrs.834

CPD Questions https://doi.org/10.1002/jmrs.870

**Table:** 

Questions	Answers
1	A
2	C
3	A
4	B
5	C

A. Fasala, M, Carr, Y. Surjan, et al. Intrafraction motion and impact of margin reduction for MR‐Linac online adaptive radiotherapy for pancreatic cancer treatments. *J Med Radiat Sci*. 2025 Mar; 72(1): 17–24. https://doi.org/10.1002/jmrs.832

CPD Questions https://doi.org/10.1002/jmrs.859

**Table:** 

Questions	Answers
1	B
2	A
3	C
4	B
5	B

### Volume 72, Special Issue 2, 2025

S. Kamarova, D. Youens, NT. Ha, et al. Demonstrating the use of population level data to investigate trends in the rate, radiation dose and cost of computed tomography across clinical groups: Are there any areas of concern? *J Med Radiat Sci*. 2025 Sep; 72(Suppl 2): S16–S30. https://doi.org/10.1002/jmrs.811

CPD Questions https://doi.org/10.1002/jmrs.829

**Table:** 

Questions	Answers
1	A
2	C
3	B
4	D
5	C

M. Lewandowska, D. Street, J. Yim, S. Jones, R. Viney. Artificial intelligence in radiation therapy treatment planning: A discrete choice experiment. *J Med Radiat Sci*. 2025 Sep; 72(Suppl 2): S42–S51. https://doi.org/10.1002/jmrs.843

CPD Questions https://doi.org/10.1002/jmrs.879

**Table:** 

Questions	Answers
1	B
2	C
3	C
4	B
5	B

### Volume 72, Issue 2, June 2025

M. Fenech, N. Mead. Collaborative use of a 3D anatomy platform to motivate and enhance anatomy learning in first‐year online medical sonography students. *J Med Radiat Sci*. 2025 Jun; 72(2): 202–208. https://doi.org/10.1002/jmrs.848

CPD Questions https://doi.org/10.1002/jmrs.886

**Table:** 

Questions	Answers
1	D
2	A
3	C
4	A
5	A

L. Rough, J. Burbery, C. Hargrave, E. Brown. An evaluation of treatment time and intrafraction motion in stereotactic body radiation therapy. *J Med Radiat Sci*. 2025 Jun; 72(2): 217–224. https://doi.org/10.1002/jmrs.861

CPD Questions https://doi.org/10.1002/jmrs.883

**Table:** 

Questions	Answers
1	B
2	C
3	B
4	D
5	A

### Volume 72, Issue 3, September 2025

Z. Moran, A. Loh, AK. Lewis, et al. Radiographer preferences for shoulder X‐ray imaging in Australia: A national survey. *J Med Radiat Sci*. 2025 Sep; 72(3): 325–332. https://doi.org/10.1002/jmrs.881

CPD Questions https://doi.org/10.1002/jmrs.894

**Table:** 

Questions	Answers
1	D
2	D
3	B
4	A
5	D

L. Wood, E. Giles, L. Cunningham, et al. Proton therapy patient selection methods and the impact of COVID‐19: A cross‐sectional international survey. *J Med Radiat Sci*. 2025 Sep; 72(3): 333–340. https://doi.org/10.1002/jmrs.885

CPD Questions https://doi.org/10.1002/jmrs.70016

**Table:** 

Questions	Answers
1	D
2	B
3	C
4	B
5	C

